# A Novel Time‐Saving Synthesis Approach for Li‐Argyrodite Superionic Conductor

**DOI:** 10.1002/advs.202301707

**Published:** 2023-05-03

**Authors:** Suk‐Ho Hwang, Seung‐Deok Seo, Dong‐Wan Kim

**Affiliations:** ^1^ School of Civil, Environmental, and architectural Engineering Korea University Seoul 02841 South Korea

**Keywords:** all‐solid‐state batteries, Li‐argyrodite, microwave‐assisted synthesis, sulfide solid electrolytes, superionic conductors

## Abstract

The wet‐chemical synthetic approach for Li‐argyrodite superionic conductors for all‐solid‐state batteries (ASSBs) is promising as it saves time, energy, and cost, while achieving scalable production. However, it faces certain commercialization issues such as byproduct generation, nucleophilic attack of the solvent, and long processing times. In this study, a facile and time‐saving microwave‐assisted wet synthesis (MW‐process) approach is proposed for Li_6_PS_5_Cl (LPSC), which is completed in 3 h at the precursor‐synthesis stage. The LPSC crystal obtained from the MW‐process presents various advantages such as fast‐PS_4_^3−^ generation, high solubility of LiCl, and low adverse effects from solvent molecules. These features help in achieving a high Li‐ion conductivity (2.79 mS cm^−1^) and low electric conductivity (1.85×10^−6^ mS cm^−1^). Furthermore, the LPSC crystal is stable when reacting with Li metal (2000 h at 0.1 mA cm^−2^) and exhibits superior cyclability with LiNi_0.6_Co_0.2_Mn_0.2_ (NCM622) (145.5 mA h g^−1^ at 0.5 C, 200 cycles with 0.12% of capacity loss per cycle). The proposed synthetic approach presents new insights into wet‐chemical engineering for sulfide‐based solid‐electrolytes (SEs), which is crucial for developing ASSBs from a commercial‐scale perspective.

## Graphical abstract

The wet‐chemical synthesis of sulfide‐based solid electrolytes is potential direction of synthetic application for all‐solid‐state batteries. A wet‐chemical synthetic method for Li‐argyrodite is newly proposed by using the microwave reactor, which has the synthetic advantages such as ultrafast PS_4_^3‐^ formation, lower solvent effect, and enhanced LiCl solubility, and high degree of sulfide/halide anion disorder.

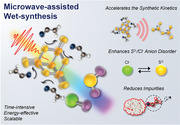

## 1 Introduction

The demand for electric vehicles and grid‐scale energy storage systems has grown rapidly in recent years, increasing the requirement for the development of batteries with high energy density and better safety features.^[^ ^1^ ^]^ The conventional lithium‐ion batteries (LIBs) cannot satisfy these requirements due to their limited energy density and risk of fire hazard which are attributed to the use of graphite anodes and liquid electrolytes (LEs), respectively.^[^ ^2^ ^]^ Consequently, ASSBs have been considered as promising alternatives due to their high volumetric energy density, better safety features, and ease of bipolar stacking with Li metal anodes.^[^ ^2^, ^3^ ^]^ The SEs are crucial in achieving these features.

Among them, Li‐containing thiophosphates such as Li_10_GeP_2_S_12_ (12 mS cm^−1^),^[^ ^1^ ^]^ Li_7_S_3_P_11_ (3.2 mS cm^−1^),^[^ ^4^ ^]^ and Li‐argyrodite type (1–24 mS cm^−1^)^[^ ^5^ ^]^ have gained considerable attention. Based on their excellent Li‐ion transport properties, which are comparable to that of LEs, along with high processability and adequate mechanical properties at room temperature, they are recognized the most up‐and‐coming successor of conventional LEs. In particular, Li‐argyrodite‐type SEs are considered to be the most feasible candidates owing to their cheap elemental cost and tunable properties (ionic conductivity and moisture‐stability), which are achieved through aliovalent substitution.^[^ ^5^ ^b–d^ ^5^, ^6^ ^]^ However, the synthetic procedure severely limits the mass‐production of SEs along with the commercialization of ASSBs.^[^ ^5^, ^7^ ^]^ Hitherto, most Li‐argyrodite‐type SEs and their derivatives have been synthesized by using a solid‐state or mechanical alloy approach, which consumes a large amount of energy and requires a long processing time.^[^ ^8^ ^]^ Therefore, the development of a simple and time‐intensive synthetic process for the realization of the commercialization of ASSBs is an immediate requirement.

The wet‐chemical synthesis method has gained considerable attention owing to its time and cost‐effectiveness in the synthesis of Li_6_PS_5_X (X = Cl, Br, and I). Since the method was first reported by the Tatsumisago group,^[^ ^9^ ^]^ several studies have been conducted based on the one or two‐step wet‐process using various solvents.^[^ ^10^ ^]^ However, these wet‐chemical methods face various drawbacks, such as by‐product generation (H_2_S gas, phosphate when using EtOH), electroconductive materials produced by co‐crystallization from nucleophilic attack of solvent, and long processing time.^[^ ^9^, ^10^, ^11^ ^]^ Extensive research has been conducted on obtaining highly pure superionic conductive materials and on developing a synthetic process with no adverse effects from the solvent to overcome these problems. However, an appropriate solution has not yet been developed. Furthermore, wet‐chemical method‐based engineering faces difficulty in controlling several reaction parameters to obtain the desired final product.

In this study, we present a facile and time‐saving microwave‐assisted approach for the synthesis of LPSC crystals. Based on a pioneering study conducted on the microwave‐assisted synthesis of lithium thiophosphate (Li_3_PS_4_),^[^ ^12^ ^]^ we successfully reduced the synthesis time for high‐quality Li‐argyrodite within 3 h by using a microwave‐assisted solvothermal process. Such a rapid reaction time has not been reported for Li‐argyrodite systems, to the best of our knowledge; it is significantly shorter than that of the conventional processes (24 h—7 d). Following annealing, LPSC SEs exhibits high ionic conductivity (2.79 mS cm^−1^, 0.26 eV of activation energy), well Cl^−^ ion substituted structure due to its high degree of S^2−^/Cl^−^ exchange, uniform particle size distribution, and Li compatibility with low overpotential (25.6 mV at 0.1 mA h cm^−2^) even after 2000 h. It also exhibits stable Li transport characteristics when assembled with LiNbO_3_‐coated NCM622 (145.5 mAh g^−1^ at 0.5 C). Therefore, this progressive method presents several advantages, such as rapid processing time, high quality of reaction products, and the feasibility of scalable production.

## 2 Results and Discussions

### 2.1 Comparison of Single‐Aprotic Solvent Synthetic Approaches

**Figure 1:**
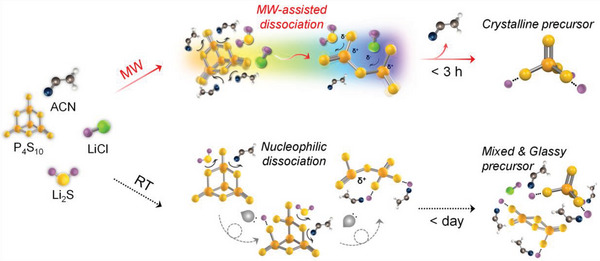
Schematic diagram of wet‐chemical synthetic approach for Li‐argyrodite via microwave‐assisted wet‐synthesis (MW‐process, top) and room‐temperature wet synthesis (RT‐process, bottom).

We prepared the LPSC crystal through MW‐process, using stoichiometrically measured Li_2_S, P_2_S_5_, and LiCl as the starting materials with an acetonitrile (ACN) solvent. These materials are also used for room‐temperature wet synthesis (RT‐process). However, the comparative illustration presented in **Figure** **1** depicts a clear difference between the MW and RT‐processes. Most wet‐process‐based LPSC synthesis method first prepare the Li_3_PS_4_ precursor to form the PS_4_^3−^ unit. The Li_3_PS_4_ precursor is typically synthesized from Li_2_S and P_4_S_10_, which consumes a large amount of time (>days) due to the typically insoluble characteristic of Li_2_S and P_2_S_5_. Particularly, P_2_S_5_ comprises a rigid adamantane‐like cage structure of P_4_S_10_, which functions as a rate limiting step during the synthesis of lithium thiophosphate SEs.^[^ ^7^, ^13^ ^]^ Consequently, almost no reaction was observed in the RT‐process when the starting materials were exposed to acetonitrile due to the low solubility of Li_2_S and P_2_S_5_ apart from the directly dissolved LiCl. However, the synthesis rate of the MW‐process is significantly higher than of the RT‐process. The instantaneous and homogeneous reactions that occur among the reactants,^[^ ^14^ ^]^ particularly the dissociation of P_4_S_10_ to reactive P_2_S_5_ (see Figure S1 and details in the Supporting Information), are aided by microwaves with rapid and deep inside heating. The directly irradiated microwaves provide uniform and rapid heating to the suspension, which accelerates the creation of uniform, well‐ordered, and isolated PS_4_^3−^ tetrahedra. This phenomenon contradicts the fact that the resultant RT‐process reaction that occurs over a longer time produces a solvent‐coordinated, mixed structure with unreacted starting materials (Li_2_S, chlorides) and amorphous PS_4_^3−^.

### 2.2 In‐/Ex‐Situ Raman Monitoring for Rapid PS_4_^3−^ Formation Mechanism of MW‐process

**Figure 2:**
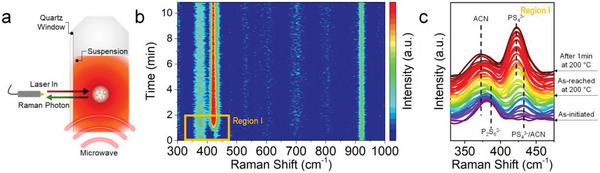
In operando monitoring results of reaction mechanism (or structural evolution) during MW process. a) Schematic diagram of combined Microwave‐Raman system. b) Time‐dependent in situ Raman spectra observed during synthesis of Li_6_PS_5_Cl (LPSC) precursor. c) Enlarged region I in (b).

The in‐situ Microwave‐Raman synthesis‐monitoring system was implemented to obtain a better understanding of the chemical structural evolution during the MW‐process, as shown in **Figure** **2**A. Figure 2B,C presents the in‐situ Raman spectroscopy results, which exhibit the synthetic mechanism and rapid reaction kinetics that occur during the PS_4_^3−^ structure formation. Prior to microwave heating, only two peaks were observed around 379 and 917 cm^−1^, indicating the C–C stretch and C–C≡N bending of ACN (Li_2_S at 369 cm^−1^ was obscured by ACN), respectively.^[^ ^12^, ^15^ ^]^ While reaching the target temperature, the peaks at ≈389 and ≈428.5 cm^−1^, which are attributed to the P_2_S_6_^2−^ anion (Li_2_S:P_2_S_5_ intermediate) and ACN‐coordinated PS_4_^3−^ tetrahedra (PS_4_^3−^/ACN complexes), respectively.^[^ ^7^, ^12^, ^13^ ^b]^ Subsequently, they are diminished and a new peak appears at 418 cm^−1^, which is attributed to the isolated PS_4_^3−^ tetrahedra.^[11a^ ^1^, ^13^ ^]^ The results demonstrate that only one minute of reaction time is required at the target temperature to achieve a stable PS_4_^3−^ structure. Furthermore, during the prolonged reaction, there is no other noticeable peak was observed, indicating that there were no additional side reactions produced by the solvent. We observed that the MW‐process rapidly and effectively obtain a stable PS_4_^3−^ polyhedron with no side reactions, which is a crucial factor in the synthesis of lithium thiophosphate. We also observed that the increasement of synthesis temperature promotes the removal of coordinated solvents from the thiophosphate structures. We conducted an ex‐situ Raman analysis at reaction temperatures of 100, 150, and 200 °C for 10 min, respectively, to determine the effect of the solvent and reaction mechanism corresponding to the synthesis temperature (Figure S2, Supporting Information). The PS_4_^3−^/ACN complex peak diminished and the PS_4_^3−^ tetrahedra peak dominated in the precursor with the increase in the temperature (see Figure S2 in the Supporting Information for details). These trends concur with the results obtained through the in‐situ Raman analysis. In summary, these results indicate that the thiophosphate polyhedron changes from meta‐thiophosphate (P_2_S_6_^2−^) to ortho‐thiophosphate (PS_4_^3−^) when coordinated with the ACN. The coordinated ACN molecules were removed from the thiophosphate structure as the reaction progressed. The nucleophilicity of the solvent, which causes co‐crystallization with the precursor, is a major problem during the solution‐based synthesis of lithium thiophosphate SEs. Notably, microwave irradiation accelerated the formation of isolated PS_4_^3−^ tetrahedra, while reducing the intervention of the solvent in the precursor.

### 2.3 Material Characterizations of Samples of MW‐process in Comparison with RT‐process

**Figure 3:**
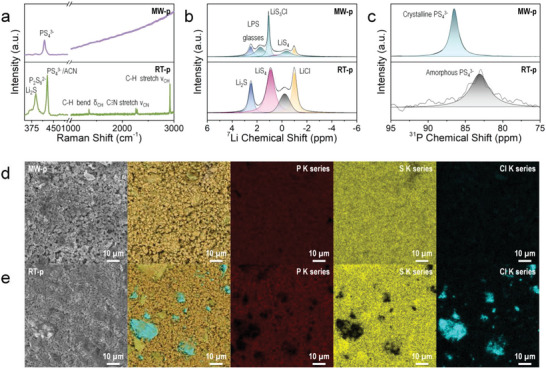
Material characterization for as‐synthesized LPSC precursor of MW‐process (MW‐p) compared to RT‐process (RT‐p). a) Raman spectra of MW‐p (top) and RT‐p (bottom). b,c) Solid‐state (b) ^7^Li and (c) ^31^P magic angle spinning (MAS) nuclear magnetic resonance (NMR) spectroscopy results of MW‐p (top) and RT‐p (bottom). d,e) Field emission scanning electron microscopy (FESEM), backscattered electron (BSE) mode image in FESEM along with layered energy dispersive X‐ray spectroscopy (EDS) map, and EDS element mapping images of (d) MW‐p and (e) RT‐p.

The LPSC precursor, which was synthesized in a much shorter period based on the microwave‐assisted synthesis method, contained more targeted materials than the RT‐process. The Raman spectra depicted in **Figure** **3**A and Figure S3 (Supporting Information) highlight the differences between the two products. The as‐synthesized LPSC precursor that was prepared through the RT‐process (RT‐p) contained PS_4_^3−^/ACN complexes (428.5 cm^−1^) along with the P_2_S_6_^2−^ anion (389 cm^−1^). The left shoulder at ≈369 cm^−1^ was also observed, which is attributed to the unreacted Li_2_S. The dried LPSC precursor that was prepared through the RT‐process (RT‐d) presented a diminished Li_2_S band, which was attributed to the progress of the reaction during the drying process, as shown in Figure S3 (bottom) in the Supporting Information. However, P_2_S_6_^2−^ anions and PS_4_^3−^/ACN complexes remained. The as‐synthesized LPSC precursor that was prepared through the MW‐process (MW‐p) primarily contains P–S bonds in the PS_4_^3−^ tetrahedra without the coordinated ACN at 418 cm^−1^, indicating that the microwave‐assisted method enables the instantaneous and homogeneous reaction of the LPSC precursor with isolated PS_4_^3−^ units within a short period, even before the annealing process. No structural changes were observed in the dried LPSC precursor that was prepared through the MW‐process (MW‐d, Figure S3, Supporting Information). Furthermore, we observed a Raman shift in the range of 1000–3000 cm^−1^ while determining the influence of solvents on the synthesis methods. The samples of RT‐process reveal the remaining solvent even after drying at ≈1355, ≈2261(2288), and ≈2911 cm^−1^, which correspond to the C–H bend, C⋮N stretch, and C–H stretch that constitute the ACN molecules.^[^ ^15^ ^]^ Conversely, the samples of MW‐process reveals the fluorescence dominated spectra, indicating that the they have different chemical structures.

X‐ray diffraction (XRD) analysis was performed to obtain the crystallographic information regarding the as‐synthesized and dried precursors. Figure S4A (MW‐process) and Figure S4B (RT‐process) in the Supporting Information depict the XRD patterns of the as‐synthesized (bottom) and dried precursors (top) obtained using two different methods. At this stage, all the samples exhibit the existence of Li_2_S (ICSD No.: 642291) and LiCl (ICSD No.: 65485), as reported previously for the Li‐argyrodite synthesis.^[10b^ ^10^, ^16^ ^]^ Some unique peaks are observed in the samples of MW‐process, which are not detected in the samples of RT‐process, exhibiting a reflection tendency similar to that previously reported for a high concentration of Cl‐containing argyrodite Li_7−_ *_x_* PS_6−_ *_x_* Cl*_x_* (*x* > 1.7).^[^ ^17^ ^]^ The result indicates the existence of already‐inserted Cl ions in the crystal structure before the annealing process, instead of the Cl ions being present separately on the outside of crystal, which helps in achieving better Cl substitution when compared to the case where they are present outside the crystalline state of LiCl.

From Raman analysis, we observed that the MW‐process is advantageous for quickly obtaining a PS_4_^3−^ tetrahedron underlying the LPSC structure. However, the Raman fluorescence and the ambiguous crystal structure in the Raman and XRD results, respectively, made it difficult to obtain more detailed information regarding the effect of the solvent and specific structural status. We conducted a more detailed analysis by combining the solid‐state magic angle spinning (MAS) nuclear magnetic resonance (NMR) spectroscopy and X‐ray photoelectron spectroscopy (XPS) analyses to better understand the local structural environments around each species and to gain insights into the structural variation and effects of the residual solvent.

We conducted solid‐state ^7^Li and ^31^P MAS NMR spectroscopy to systematically identify the local compositional effects of the products, especially for invisible Cl ions in the structure not observed in Raman spectroscopy, which is crucial for synthesizing S^2−^/Cl^−^ disordered argyrodite structures. The ^7^Li MAS NMR spectra of MW‐p primarily exhibit peaks at 1.08 ppm with a lower frequency shoulder −0.35 ppm, which is attributed to the Li(S_3_Cl) and LiS_4_ tetrahedra, respectively, as shown in Figure 3B (top).^[^ ^18^ ^]^ This result corresponds to the X‐ray diffraction patterns of MW‐p depicted in Figure S4A (Supporting Information), where the Cl ions are introduced by constructing a tetrahedral framework. Furthermore, we observed a peak at 1.70 ppm, which corresponds to the previously reported (Li_2_S)*_x_*–(P_2_S_5_)_1−_ *_x_* glasses with Li_2_S content (*x* > 0.66) (LPS glasses, Figure 3B). The ^31^P MAS NMR spectra exhibit a peak at 86.6 ppm, which is attributed to the crystalline PS_4_^3−^ structure, as shown in Figure 3B (top).^[^ ^19^ ^]^ These results indicate that a well‐ordered PS_4_^3−^ structure linked with Cl ions in the form of the Li(S_3_Cl) tetrahedra was created after only the MW reaction, which is denoted as the LPSC intermediate. Additionally, small amounts of Li_2_S and LiCl were also observed at 2.50 and −0.99 ppm, respectively.^[^ ^19d^ ^]^ There were no significant changes observed in the ^7^Li and ^31^P NMR spectra of the MW‐d (Figure S5A,B, top, Supporting Information) after the low temperature drying process.

However, these results differed from those of the RT‐processes. The ^7^Li MAS NMR spectra of RT‐p exhibited large amounts of Li_2_S and LiCl, as shown in Figure 3B (bottom). Additionally, the LiS_4_ tetrahedron in the Li_3_PS_4_ structure was observed at 0.92 ppm in Figure 3B (bottom), whose value is lower than that of the previously reported value for Li_3_PS_4_ due to adjacent ACN molecules.^[^ ^19^ ^d]^ This trend is also observed in the ^31^P MAS NMR of the RT‐p (Figure 3C, bottom). The disordered PS_4_^3−^ was observed at 83.12 ppm slightly shifted from reported the amorphous PS_4_^3−^.^[19c^ ^19^, ^20^ ^]^ After drying process in Figure S5A (bottom) (Supporting Information), the LiCl (at 0.99 ppm) peak was reduced and the peak at 0.19 ppm in Figure 3B (bottom) became distinguishable into Li_3_PS_4_ and LPSC intermediate, at 0.43 and −0.35 ppm, respectively.^[^ ^19^ ^d]^ Furthermore, in the ^7^Li and ^31^P MAS NMR spectra of RT‐d in (Figure S5A,B, bottom, Supporting Information), the peaks were slightly shifted to lower and higher field to 1.01 and 82.94 ppm, respectively. According to the previously reported *β*‑Li_3_PS_4_ formation mechanism, these results originate from changes in the lithium and phosphorous environments due to variations in the P–S bond distance.^[^ ^21^ ^]^

These differences are conspicuous in the field‐emission scanning electron microscopy (FESEM) and energy dispersive spectroscopy (EDS) images. As shown in Figure 3D,E (MW‐p and RT‐p, respectively), the chemical distribution of MW‐p was more uniform than that of RT‐p. RT‐p shows a large amount of unreacted Cl compounds, which shows that the MW process is advantageous for increasing the Cl ion solubility, resulting in high anion site disorder of the final products (discussed below).

**Figure 4:**
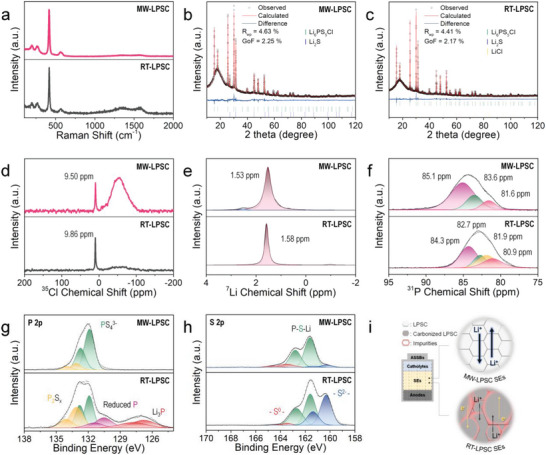
Physical and chemical characterization of Li‐argyrodite LPSC. a,b) Rietveld refinement of XRD pattern of Li‐argyrodite, which was synthesized through (a) MW‐process (MW‐LPSC) and (b) RT‐process (RT‐LPSC). c) Raman spectra of MW‐LPSC (top) and RT‐LPSC (bottom). d–f) Solid‐state (D) ^7^Li, (E) ^31^P, and (F) ^35^Cl MAS NMR spectroscopy results of MW‐LPSC (top) and RT‐LPSC (bottom). g,h) (g) S 2p and (h) P 2p spectra of MW‐LPSC (top) and RT‐LPSC (bottom). i) Schematic diagram of the final product of MW‐process and RT‐process.

As mentioned above, the structural differences and interactions with solvents were confirmed in the as‐synthesized samples. To observe effect of co‐crystallized solvent after the annealing process we performed Raman spectroscopy analysis (**Figure** **4**A). Both samples contain PS_4_^3−^ tetrahedra, constructing an argyrodite structure at 418 cm^−1^. However, Li‐argyrodite LPSC that was synthesized thorough the RT‐process (RT‐LPSC) represents a greater number of peaks at ≈1328 and ≈1573 cm^−1^ compared to Li‐argyrodite LPSC that was synthesized through the MW‐process (MW‐LPSC), which were assigned to the D and G bands due to the carbonized residual solvents, respectively.^[^ ^22^ ^]^ Then, we further analyzed the N 1s XPS to examine the interaction of the nitrile group with the precursor after soft sputtering at 0.5 kV for 90 s to prevent misinterpretation due to inevitable contamination. The MW‐p exhibits N atoms in nitrile group at 399.4 eV assigned to residual ACN and its disappearance after the drying step, as shown in Figure S6A (MW‐process) in the Supporting Information.^[^ ^23^ ^]^ Conversely, the residual ACN in the RT‐process (Figure S6B, Supporting Information) does not disappear after the drying step, and even creates a new bonding with Li after annealing (Li–N, 399 eV), indicating the strong coordination of the residual solvent in the crystal structure (co‐crystallization).^[^ ^24^ ^]^

We performed further characterizations after annealing to determine if these differences affect the results of the two different methods. XRD analysis and Rietveld refinement were performed (Figure 4B,C; Tables S1 and S2, Supporting Information) after the annealing process to analyze the purity and crystallinity of Li‐argyrodite LPSC. The XRD patterns of the MW‐LPSC and RT‐LPSC primarily exhibit an argyrodite structure with a cubic LPSC phase (F‐43m). However, the RT‐LPSC exhibited more impurities such as Li_2_S (2.5 wt%) and LiCl (3.98 wt%) than the MW‐LPSC, which has only Li_2_S (5.10 wt%), in the Rietveld refinement results. These results demonstrate that the microwave helps the Cl ion enter the argyrodite structure, which presents a higher purity of the LPSC than the RT‐process. Furthermore, the Cl ion in the MW‐LPSC occupy more the Wyckoff 4a site than the RT‐LPSC, which is also shown in the solid‐state ^35^Cl MAS NMR spectroscopy results. As shown in the Figure 4D, the solid‐state ^35^Cl MAS NMR spectroscopy results illustrated that the degree of anion disorder (S^2−^/Cl^−^) was derived from the facilitated Cl incorporation through the MW‐process, which is a decisive factor in the Li^+^ ion conductivity in the Li‐argyrodite LPSC structure. MW‐LPSC exhibits a broader signal at ≈53.81 ppm than RT‐LPSC, indicating that the MW‐process induces the anion site disorder between the Wyckoff 4a and 4d sites in the argyrodite structure.^[^ ^25^ ^]^ Furthermore, MW‐LPSC and RT‐LPSC exhibit peaks at 9.50 and 9.86 ppm, respectively.^[25a^ ^25^, ^26^ ^]^ The peak of MW‐LPSC is shifted to a higher field region than RT‐LPSC, which is caused by the large amount of modified anion charges in the tetrahedral framework. These results concur well with the crystallographic data presented in Tables S1 and S2 in the Supporting Information. Based on the in/ex‐situ Raman spectroscopy and XRD refinement results, the microwave accelerates the formation of the isolated PS_4_^3−^ unit originating from the argyrodite structure without solvent intervention, while also increasing the anion site disorder in the final products.

Furthermore, the ^7^Li MAS NMR spectra of MW‐LPSC exhibits the peak at 1.53 ppm, corresponds to the PS_4_^3−^ structure in the Li‐argyrodite LPSC (Figure 4E, top).^[^ ^26^ ^]^ The RT‐LPSC exhibits a slightly shifted peak at 1.58 ppm (Figure 4E, bottom). These differences correspond to the electron density around the Li atom based on the degree of S^2−^/Cl^−^ site disorder, which concurs with the ^31^P MAS NMR results (Figure 4F). The MW‐LPSC exhibits peaks at 85.1, 83.6, and 81.6, which were assigned to PS_3_Cl^2−^, PS_2_Cl_2_^1−^, and PSCl_3_, respectively (Figure 4F, top).^[25b^ ^25^, ^26^ ^]^ Conversely, the spectra of RT‐LPSC shifted to a higher field (Figure 4F, bottom), which is caused by the lower degree of S^2−^/Cl^−^ site disorder than MW‐LPSC due to (causing) the decreased electron distribution surrounding the P sites. These results concurred well with the ^35^Cl MAS NMR results presented in Figure 4D. Additionally, RT‐LPSC depicts amorphous thiophosphate species at 81.9 ppm.^[^ ^19^ ^d]^ Consequently, the site exchange between S^2−^ and Cl^−^ ions in the PS_4_^3−^ framework changes the electron distribution around the Li, P, and Cl sites, leading to the structural distortion and weakening of the Li^+^–host interaction, which is a key factor of the Li^+^ ion mobility in the Li‐argyrodite LPSC structure. Therefore, we confirmed that a larger number of coordinated Cl ions in the precursor induced a higher degree of anion site disorder in the LPSC structure.

The MW‐process does not exhibit any chemical state changes in the Li 1s and Cl 2p XPS spectra, maintaining the Li–S bond at 55.5 eV and the Cl^−^ ion at 198.8 (Cl 2p3/2) and 200.3 (Cl 2p1/2) eV doublets, as shown in Figure S7A,B (Supporting Information), indicating that MW‐p has similar chemical environment with the Li‐argyrodite LPSC.^[^ ^27^ ^]^ However, the RT‐process exhibits a slightly different chemical state in the Li 1s spectra, as shown in Figure S7C in the Supporting Information. Before the annealing process, RT‐p and RT‐d exhibited an environment similar to that of the samples of MW‐process in the Li 1s spectra (Figure S7C, bottom and middle, Supporting Information). After the annealing process, Li_2_S appeared at 54.6 eV in the RT‐LPSC (Figure S7C, top, Supporting Information).^[^ ^28^ ^]^ The RT‐process did not show any changes in the Cl 2p spectra along with the MW‐process, as shown in Figure S7D in the Supporting Information. These trends were observed in the S 2p and P 2p spectra of the resultants. In the P 2p spectra (Figure 4G, top), the MW‐LPSC exhibits the main doublets at 131.8 (P 2p3/2) and 132.7 (P 2p1/2) eV, which are attributed to the P‐S bond in the PS_4_^3−^ tetrahedra.^[^ ^27^ ^b]^ Additionally, small doublets were also observed at 133.1 (P 2p3/2) and 134 (P 2p1/2), which are caused by the oxidized phosphorous species such as P_2_S_5_ or P_2_S*_x_* at the surface.^[^ ^29^ ^]^ The S 2p spectra of the MW‐process exhibit the PS_4_^3−^ doublets at 161.6 (S 2p3/2) and 162.5 (S 2p1/2) eV with the left side shoulder, which attributed to the P–S–S–P structures in the P_2_S_6_^2−^ unit formed by the oxidized sulfur species at the electrolyte surface at 163.4 (S 2p3/2) and 164.5 eV (S 2p1/2), which correspond to the P 2p spectra.^[^ ^29^ ^]^ The peaks at 160.3 (S 2p3/2) and 161.3 eV (S 2p1/2) were observed in the MW‐LPSC (Figure 4H, top), which attribute to the S^2−^ ion in the Li_2_S.^[^ ^30^ ^]^ Similarly, these chemical states remain unchanged after further annealing (Figures S8A,B in the Supporting Information except for S^2−^ ion in the Li_2_S). These results demonstrate that the LPSC precursor synthesized by the MW‐process has an environment that is very similar to that of the Li‐argyrodite LPSC.

However, the RT‐process exhibited a relatively larger number of impurities and impacts due to the unremoved solvents than the MW‐process at the surfaces. The P 2p and S 2p spectra depicted in Figure S8C,D (Supporting Information) demonstrate that the reaction progressed more during the drying process. The incomplete species such as P_2_S*_x_*, reduced phosphorus species (130.5 (P 2p3/2) and 131.4 (P 2p1/2) eV),^[^ ^31^ ^]^ and Li_2_S were diminished, which corresponds to the Raman and NMR spectroscopy results (Figure 3A,B; Figures S3 and S5A, Supporting Information). In the P 2p spectra, the RT‐LPSC shows many impurities, such as Li_3_P (126.8 (P 2p3/2) and 127.8 (P 2p1/2) eV) and reduced phosphorous species (Figure 4G, bottom).^[^ ^31^ ^]^ Furthermore, the RT‐LPSC also exhibits extremely high intensity of S^2−^ ion in the Li_2_S at 160.3 (S 2p3/2) and 161.3 (S 2p1/2) eV, as shown in the S 2p spectra (Figure 4H, bottom), which is attributed to the reduced phosphorous species at the surface. These impurities were formed by the interposed ACN molecules in the crystal structure. The nucleophilic nitrile group strongly interacts with the positively charged Li atoms, generating a locally nonstoichiometric phase in the LPSC precursor. After the annealing process, it is co‐crystallized with the precursor, forming several impurities, such as reduced P, Li_3_P, Li_2_S, Li–N bonds, and carbonized solvents, which produce resistance at the grain boundary and impede ionic motion in the lattice structure and electron conduction in the SEs (discussed below). Furthermore, we examine the morphology and elemental distribution using FESEM and EDS. The resultant of the MW‐process exhibited a more uniform size than the RT‐process (Figure S9B, Supporting Information), as shown in Figure S9A in the Supporting Information. This demonstrates that the MW process can increase the purity and uniformity of the synthesis. Figure 4I depicts the results of the two different methods.

### 2.4 Transport Properties of SEs

**Figure 5:**
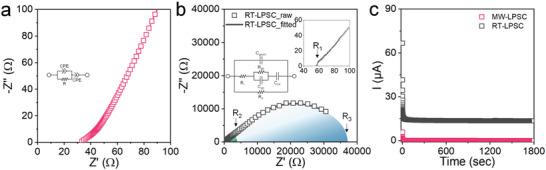
Electrochemical characterization of MW‐LPSC and RT‐LPSC SEs. a) Nyquist plot of MW‐LPSC frequency range from 3 MHz to 10 mHz at room temperature (inset: equivalent circuit used to fit the measurement of MW‐LPSC). b) Nyquist plot of the RT‐LPSC frequency range from 3 MHz to 10 mHz at room temperature (inset: the equivalent circuit used to fit the measurement of RT‐LPSC and enlarged the Nyquist plot). c) Direct‐current polarization profiles of MW‐LPSC (pink) and RT‐LPSC (gray) with applied voltage of 0.5 V.

We assembled a Ti/SEs/Ti customized symmetric cell in an Ar‐filled atmosphere to evaluate the ionic and electrical conductivities of the SEs synthesized by the MW and RT‐process. **Figure** **5**A depicts the Nyquist plots of the MW‐LPSC SEs measured using the electrochemical impedance spectroscopy (EIS) at 25 °C. The Nyquist plots were fitted with a constant phase element (CPE) in series with the parallel CPE/resistance of the blocking electrode (Figure 5A, inset). However, only the tail of the ion‐blocking electrode, which accounted for the Li^+^ diffusion, was fitted since the CPE/resistor was shifted to higher frequencies at 25 °C. Consequently, it was difficult to distinguish the bulk resistance and grain boundaries. The MW‐LPSC SEs present a resistance of 33.3 Ω at room temperature, which was converted to total conductivities of 2.79 mS cm^−1^. Furthermore, we obtained the Arrhenius diagrams by using the temperature‐dependent EIS to evaluate the activation energy barrier for Li^+^ conduction at temperatures ranging from 25 to 80 °C (Figure S10, Supporting Information). The calculated activation energy of the MW‐LPSC SEs is 0.26 eV, which is slightly lower than the previous reported values for the Li‐argyrodite LPSC.^[^ ^32^ ^]^ We thus demonstrated that the MW‐process presents effective solution‐based engineering for argyrodite, which forms a pure ionic conductor within a very short period. Subsequently, the electrical conductivity was measured through direct current (DC) polarization by applying a DC voltage of 0.5 V (Figure 5C, pink). The DC electrical conductivity was calculated to be 1.85 × 10^−6^ mS cm^−1^ for the MW‐LPSC SEs from a stabilized current, which is six orders of magnitude lower than the ionic conductivity.

However, the RT‐LPSC SEs exhibit two semicircles at middle and low‐frequencies, as shown in Figure 5B, which are attributed to the grain boundary resistance (*R*_gb_) and electrical resistance (*R*_e_) caused by the impurities at the SEs surfaces and internal carbon species in the ionic conductor, respectively. If the ion transport species passing through a solid are disturbed by an internal interface, such as a grain boundary, the additional ionic impedance behaves as an internal ionic resistance connected in series.^[^ ^33^ ^]^ Furthermore, the semicircle at the low frequency is configured in parallel with *R*_e_ and ionic path resistance (*R*_i_). The inset of Figure 5B depicts these equivalent circuits. The resistances, *R*_i_, *R*_gb_, and *R*_e_, were calculated as follows

(1) $$R_{i}=R_{3}R_{1}/\left(R_{3}-R_{1}\right)\left(R_{3}=R_{e}\right)$$

(2) $$R_{\mathrm{gb}}=R_{2}-R_{1}$$

where *R*_1,_ *R*_2_, and *R*_3_ represent the x intercepts in the high, middle, and low‐frequency regions, respectively. Additionally, *R*_3_ denotes the *R*_e_. The ionic and electrical conductivities were derived from the above equation. Table S3 in the Supporting Information lists the values of the calculated resistances and conductivities. The bulk ionic conductivity of RT‐LPSC SEs is 1.6 mS cm^−1^, which is lower than that of the MW‐LPSC SEs due to their limited anion site disorder. Furthermore, the ionic conductivity was disrupted by a discontinuous lattice in the SEs due to interfacial impurities, which led to additional resistance behavior by the grain boundary, generating an unwanted ionic conduction of 1.87 × 10^−2^ mS cm^−1^. The electrical conductivity was calculated to be 2.38 × 10^−3^ mS cm^−1^, which is approximately equal to the value of 2.48 × 10^−3^ mS cm^−1^ that was derived using the DC polarization measurements (Figure 5C, gray). Its value is a 1000‐fold higher in magnitude than that of the MW‐LPSC SEs, which produces a low open current voltage (OCV) (discussed below). These electrochemical results concur well with the experimental results that are affected by undesirable impurities at the surface and the carbon species in the final products (Figure 4; Figure S6 and S7, Supporting Information).

We assembled the half‐cells by using MW‐LPSC and RT‐LPSC as SEs to estimate the Li compatibility of ASSBs. First, we performed cyclic voltammetry (CV) and galvanostatic charge/discharge (GCD) symmetric cell tests to evaluate the interfacial stability. For the CV tests, Ti/SE/Li asymmetric cells were employed at a scan rate of 5 mV s^−1^ and a voltage range of −0.5 to 5 V versus Li^+^/Li. The Ti/MW‐LPSC SEs/Li cell (pink) exhibited more effective Li striping/plating behavior than the Ti/RT‐LPSC SEs/Li cell (gray) in the SEs asymmetric test without side reactions above 0 V versus Li^+^/Li, as shown in Figure S11 in the Supporting Information. The CV results indicate that the presence of internal transverse elements, such as electron leakage and grain boundary resistance, impedes the Li striping/plating and generates undesirable electrochemical redox reactions. Furthermore, we performed GCD measurements of the Li|SEs|Li symmetric cell to evaluate the reversible stability of the metallic Li anodes. The initial overpotential of the Li|RT‐LPSC SEs|Li (gray) and Li|MW‐LPSC SEs|Li (pink) symmetric cell presents 11.08 and 11.15 mV at a fixed areal capacity of 0.1 mA cm^−2^, respectively, as shown in Figure S12 in the Supporting Information. There was no significant difference in the initial values due to electron leakage in the RT‐LPSC. However, the Li|RT‐LPSC SEs|Li symmetric cell exhibited a gradual voltage decrease after 78 h, which is attributed to the short circuit in the cell (Figure S12, inset, Supporting Information). Conversely, the Li|MW‐LPSC SEs|Li symmetric cell could transport a stable cycle over 2000 h without a drastic increase in the overpotential (only 25.6 mV). These differences concur with the EIS results, indicating that the RT‐LPSC SEs have a larger grain boundary resistance and internal conductive carbon species (impeded Li^+^ conduction and unstable interfacial stability).

### 2.5 Electrochemical Performances of MW‐LPSC for ASSBs

**Figure 6:**
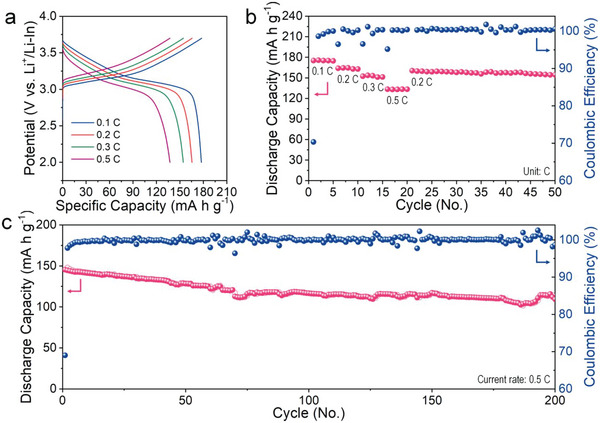
Cycling performances of ASSBs with MW‐LPSC SEs. a) Galvanostatic charge/discharge voltage curve at 0.1, 0.2, 0.3, and 0.5 C (1 C = 180 mA g^−1^). b) Discharge capacity and Coulombic efficiency as a function of cycle number at various C rates. c) Long‐term stability test at 0.5 C.

We assembled a customized stainless‐steel framework cell that was subjected to a constant pressure of 70 MPa in an Ar‐filled atmosphere to evaluate the electrochemical performance of these SEs. We employed a LiNbO_3_ – coated NCM622/SE/conductive carbon (Super P)‐mixed catholyte and Li–In alloy powder as an anode to avoid the decomposition SEs and to maintain a constant redox potential, respectively. **Figure** **6**A depicts the GCD curves at various scan rates of 0.1, 0.2, 0.3, 0.5 C when the MW‐LPSC was used as the SEs. The MW‐LPSC cell achieved superior specific capacities of 175.2, 164, 152.4, and 133.5 mA h g^−1^ at 0.1, 0.2, 0.3, and 0.5 C, respectively. When the scan rate returned to 0.1 C, the discharge capacity returned to 160.5 mA h g^−1^, retaining over 50th cycles with a capacity retention of 96.2% (Figure 6B). Conversely, the RT‐LPSC cell exhibits a lower OCV of 1.09 V when compared to the MW‐LPSC cell (≈1.7 V) depicted in Figure S13A,B (Supporting Information), which was originated by electron leakage because of co‐crystallized ACN in SEs as explained earlier. Consequently, the initial charge curve of the RT‐LPSC cell exhibits an irreversible capacity due to the deterioration of the electrolytes. These results indicate that the residual solvent in the SEs affects the electrochemical characteristics. Figure 6C depicts the long‐term cycling test, in which the MW‐LPSC SEs also exhibited excellent specific capacity (145.5 mA h g^−1^ at 0.5 C) and capacity retention 200 cycles with 0.12% of capacity loss per cycle. Furthermore, our ASSBs performances are superior to that of Li‐argyrodite SEs via typical wet‐chemical synthesis using layered‐structured cathode materials such as LiNi_1−_ *_x_*_−_ *_y_* Co*_x_* Mn*_y_* O_2_ (Table S4, Supporting Information).

## 3 Conclusion

In summary, we proposed a novel wet‐chemical synthesis method to prepare a Li‐argyrodite‐type superionic conductor by using the MW‐process, which has been reported for the first time. The MW‐process induces an instantaneous and homogeneous reaction for the LPSC precursor, which presents: (1) rapid formation of the unit PS_4_^3−^ structure, (2) effective accommodation of Cl^−^ ions, and (3) significantly suppressed solvent intervention. Furthermore, we systematically analyzed the reaction pathway and merits of the MW process by using in‐situ characterization techniques. Consequently, the final products obtained exhibited high purity, uniformity, and Li^+^‐ion conductivity with a large amount of Cl^−^ substituents when compared to those obtained using the RT‐process, which effectively functions as a pure Li‐ion conductor (2.79 mS cm^−1^). The ASSBs exhibited remarkable performance in rate capability with long‐term stability (145.5 mA h g^−1^ at 0.5C and 200 cycles with 0.12% capacity loss per cycle). Furthermore, the Li symmetric cell also exhibited an excellent interfacial stability of 0.1 mA cm^−1^ with over 2000 h. This synthesis approach presents various insights and forms the basis for the wet‐chemical engineering of sulfide‐based SEs, which is crucial for the commercialization of ASSBs.

## Conflict of Interest

The authors declare no conflict of interest.

## Supporting information

Supporting Information

## Data Availability

The data that support the findings of this study are available in the supplementary material of this article.
