# Supplementary material for: A Novel Time‐Saving Synthesis Approach for Li‐Argyrodite Superionic Conductor
Source: Adv Sci (Weinh). 2023 May 3;10(22):2301707. doi: 10.1002/advs.202301707 (PMC10401185; doi:10.1002/advs.202301707)
Supplement: Supplementary file 1 — Supporting Information [file ADVS-10-2301707-s001.pdf]

ADVANCED SCIENCE
Open Access

Supporting Information

for *Adv. Sci.*, DOI 10.1002/advs.202301707

A Novel Time-Saving Synthesis Approach for Li-Argyrodite Superionic Conductor

*Suk-Ho Hwang, Seung-Deok Seo and Dong-Wan Kim**

Supporting Information

A Novel Time-saving Synthesis Approach for Li-argyrodite Superionic Conductor

*Suk Ho Hwang, Seung-Deok Seo, and Dong-Wan Kim**

Experimental Details

1. Material Synthesis

Li-argyrodite Li_6PS_5Cl (LPSC) were synthesized through microwave-assisted wet synthesis (MW-process) and room-temperature wet synthesis (RT-process). Lithium sulfide (Li_2S, Sigma-Aldrich, 99.98%) and phosphorus pentasulfide (P_4S_{10}, Sigma-Aldrich, 99%) with a stoichiometric molar ratio of the target materials were dispersed in anhydrous ACN in an Ar-filled glove box. For a MW-process, the suspension was transferred to microwave reactor (Monowave 400 R, Anton-Paar, Switzerland). It was then heated to 200°C through microwave irradiation and stirred at 1200 rpm for 3h using 30.2 W of average power, 786 W of maximum power and 94.8 Wh of overall energy. The obtained suspension was transferred to an Ar-filled glove box and the solvent was evaporated at room-temperature to investigate the as-synthesized status of precursor. Subsequently, the obtained powder was vacuum-dried at 140°C for 3h to thoroughly remove the residual solvent. The dried powder was then sealed in Ar-filled quartz ampoule and sintered at 550°C for 5h in a furnace. For the RT-process, the suspension was stirred at room temperature at 1200 rpm. The remaining process is identical to that of the MW-process.

2. Material Characterization

The crystal structure of the as-synthesized and dried products were defined through X-ray diffraction (XRD, Miniflex, Rigaku, Japan) with a step size of 0.02° and 1 min per step from 10°-70° (2θ). Rietveld refinement analysis was conducted with a step size of 0.02° and 1 min per step from 10°-120° (2θ) by using an X-ray powder diffractometer (D8 Advance, Bruker, USA). All the samples were placed on the (911)-oriented silicon zero-diffraction plate; the air-tight sample holder was sealed with polyamide Kapton film, and the XRD patterns were then measured using a monochromatic Cu Kα radiation source (λ = 1.5406 Å). Raman

spectroscopy (LabRam ARAMIS IR2, HORIBA JOBIN YVON, France) and X-ray photoelectron spectroscopy (XPS, K-Alpha +, Thermo Fisher, USA) were employed to verify the chemical structure and valence state. Raman spectroscopy was performed using a cover glass that was sealed on both sides using vacuum grease and Kapton tape to prevent air exposure. The *in-situ* Raman spectroscopy (Cora 5001, Anton-Paar, Switzerland) was performed in conjunction with Monowave 400 R. XPS analysis was performed under an Ar atmosphere using a transfer vessel from a glove box, and the powder was softly sputtered at 0.5 kV for 90 s to clean the inevitable contaminations that occur at the surface. Field emission scanning electron microscopy (FESEM, Regulus 8230, Hitachi, Japan) and energy dispersive spectroscopy (EDS, Ultim Max 170, Oxford Instruments, United Kingdom) were employed to analyze the morphology and presence of each element of the argyrodite SEs. The nuclear magnetic resonance (NMR) spectroscopy (VNMRS 600, Varian, USA) was performed using a 1.6 mm zirconia probes at a spinning rate of 35 kHz to better understand the chemical structure of SEs, solid-state ^{7}Li, ^{31}P, and ^{35}Cl single-pulse magic angle spinning (MAS). The ^{7}Li, ^{31}P, and ^{35}Cl MAS NMR spectra were measured under a magnetic field of 14.1 T and Larmor frequencies of 233.1, 242.8, and 58.7 MHz, respectively. The ^{7}Li MAS NMR spectra were measured under a $\pi/8$ pulse length of 0.35 μs, 16 transients, and a relaxation delay of 10 s. The ^{31}P MAS NMR spectra were measured under a $\pi/2$ pulse length of 1.44 μs, 64 transients, and a relaxation delay of 20 s. The ^{35}Cl MAS NMR spectra were measured under a $\pi/2$ pulse length of 2.55 μs, 1024 transients, and a relaxation delay of 0.2 s. The chemical shifts for the ^{7}Li, ^{31}P, and ^{35}Cl MAS NMR spectra were calibrated to 1M LiCl, 85% H_3PO_4, and 1M NaCl solution at 0 ppm.

3. Electrochemical Characterization and All-Solid-State Batteries (ASSBs) Cell Assembly

An electrochemical measurement system (Compactstat, Ivium Technologies, Netherlands) was used to determine the ionic and electronic conductivity. The electrochemical impedence

spectroscopy (EIS) and direct current (DC) polarization were conducted using pelletized SEs that were fabricated by cold-pressing ~100 mg of powder in a 10 mm cylindrical polyether ether ketone (PEEK) mold between the two Ti rods at 370 MPa for 3 min using a uniaxial hydraulic press. The EIS (frequency range: 3MHz – 0.01 Hz; AC amplitude: 10 mV) within a temperature range of 25°C to 80°C and DC polarization (applying voltage: 0.5 V for 30 min) at 25°C were measured using a custom-made stainless steel framework cell, which was subjected to a constant pressure of 70 MPa. Galvanostatic and cyclic voltammetry (CV) tests were performed by using an automatic battery cycler (WBCS 3000, Wonatech, Korea). The galvanostatic tests of the symmetric cell (Li|SEs|Li) were performed at an areal current density of 0.1 mA cm^{-2} and cut-off capacity of 0.1 mAh cm^{-2} at 1 MPa. CV tests were performed to evaluate the electrochemical stability window. The cell configuration of Li|SEs|Ti was determined for the CV test. For the fabrication of Li|SEs|Ti, 100 mg of SEs were placed into the PEEK mold and cold-pressed at 370 MPa for 3 min. The Li metal foil was used as the counter/reference electrode. The Li|SEs|Ti composites were pressed at 1 MPa in a custom-made stainless steel framework cell, and the CV measurements were performed for a voltage window (-0.5 V-5 V vs. Li/Li^+) at a constant scan rate (50 mV s^{-1}). The single-crystal NCM622 (MSE Supplies, US) was used as the active material to measure the performance of the ASSBs. Subsequently, the catholyte was fabricated by mixing an agate mortar for 15 min with an SE, an active material, and a conductive carbon additive (Super P, MMM carbon, Belgium) in a weight ratio of 70:30:3. For the fabrication of full-cells for ASSBs, 70 mg of SEs were cold-pressed using PEEK mold at 370 MPa for 3 min, and then 10 mg of the catholytes were spread over side of SEs and pelletized at 370 MPa for 3 min. The areal loading mass of catholytes is 6.80 mg cm^{-2}, resulting in total cathode thickness of 70 μm. To fabricate the anode material, lithium (NEBA, 99.9%) and indium (Sigma-Aldrich, 99.99%) powders were mixed in a molar ratio of 0.5:1, resulting in total anode mass of 68 mg and thickness of 110 μm. The Li/In|SEs|catholyte composites were assembled by uniaxial

pressing at 70 MPa, and the pressure was maintained. The assembled cells were measured via a GCD cycling test at various current rates (1 C = 180 mA g^{-1}) within a voltage window of 2.0-3.7 V (vs. Li/Li^{+}). All the characterization and cell assembly procedures were conducted under an Ar atmosphere at room-temperature.

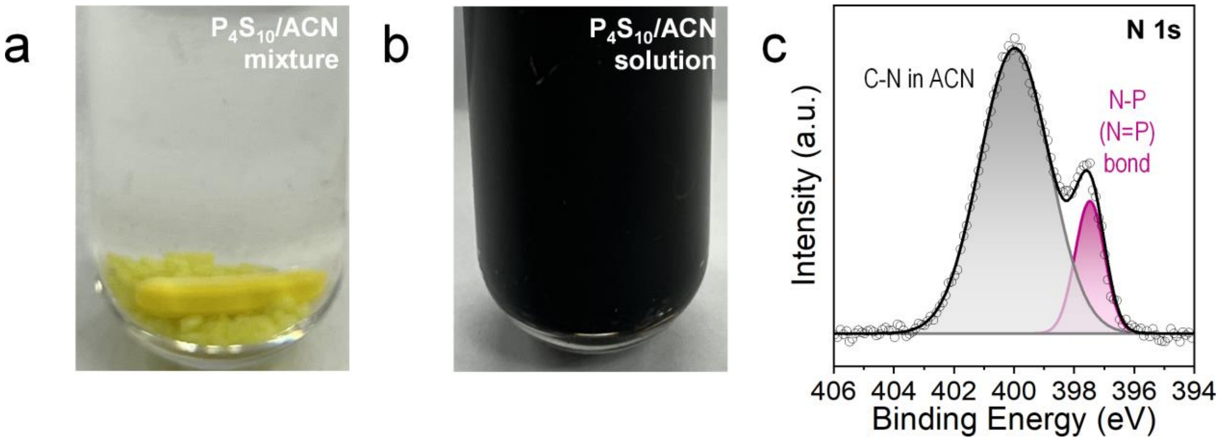

Figure S1. Microwave-treatment results of P_4S_{10} mixture with acetonitrile (P_4S_{10}/ACN). (a) Photographs of P_4S_{10}/ACN. (b) Solution of microwave-treated P_4S_{10}/ACN mixture at 200°C for 10min.

(c) N 1s XPS spectra of dried powder of microwave-treated P_4S_{10}/ACN solution.

Figure. S1 presents the photographs of the P_4S_{10} mixture with ACN before and after the microwave treatment. Prior to the treatment, the mixture was suspended without any change. However, microwaves easily dissociated the P_4S_{10} within 10 min at 200°C, producing a dark purple solution. To analyze the interaction of P_4S_{10} with ACN, we first dried the solution and then performed XPS analysis using the dried P_4S_{10}/ACN powders. The N 1s spectra depicts a cyano group (C≡N) in the ACN and N-P bonding at 399.8 and 397.5 eV, respectively, as shown in Figure S1.[1] A new bonding was formed between phosphorous and nitrogen similar to the previously reported P_2S_5/pyridine complexes.[2] Therefore, we confirmed that dielectric (microwave) heating aided in the dissociation of P_4S_{10} into P_2S_5/ACN complexes.

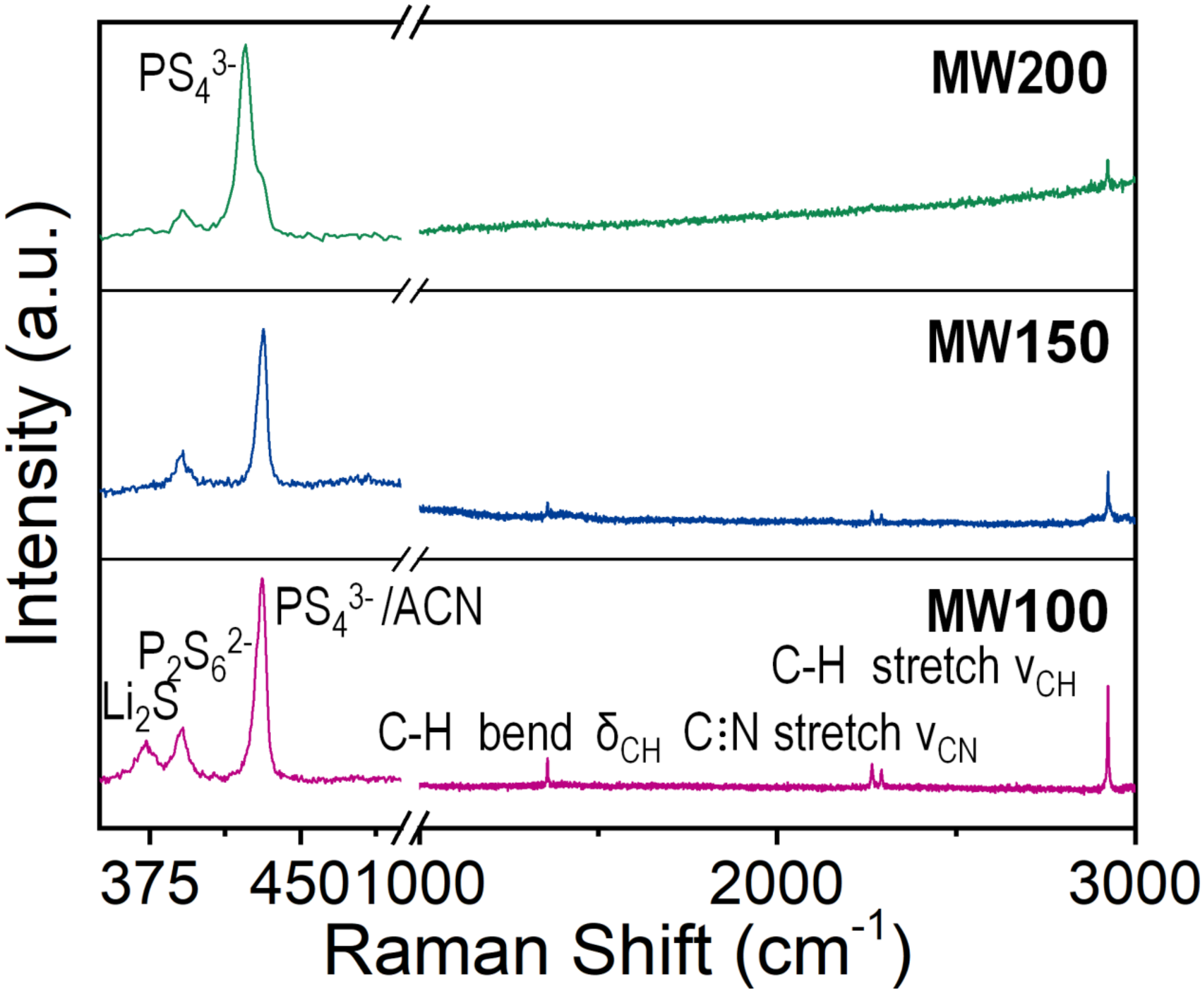

Figure S2. *Ex-situ* Raman spectra of temperature-dependent MW-process for LPSC precursor at 100°C (MW100, bottom), 150°C (MW150, middle), and 200°C (MW200. top) for 10 min, respectively

As the reaction progresses and with the increase in the synthesis temperature, the Li_2S, $P_2S_6^{2-}$, and PS_4^{3-}/ACN peaks disappear at 369 cm^{-1}, 389 cm^{-1}, and 428.5 cm^{-1}, respectively.[3] Furthermore, the peaks at ~1355 cm^{-1}, ~2261(2288) cm^{-1}, and ~2911 cm^{-1} decrease, corresponding to the C-H bend, C⫶N stretch, and C-H stretch that constitute the ACN molecules.[4]

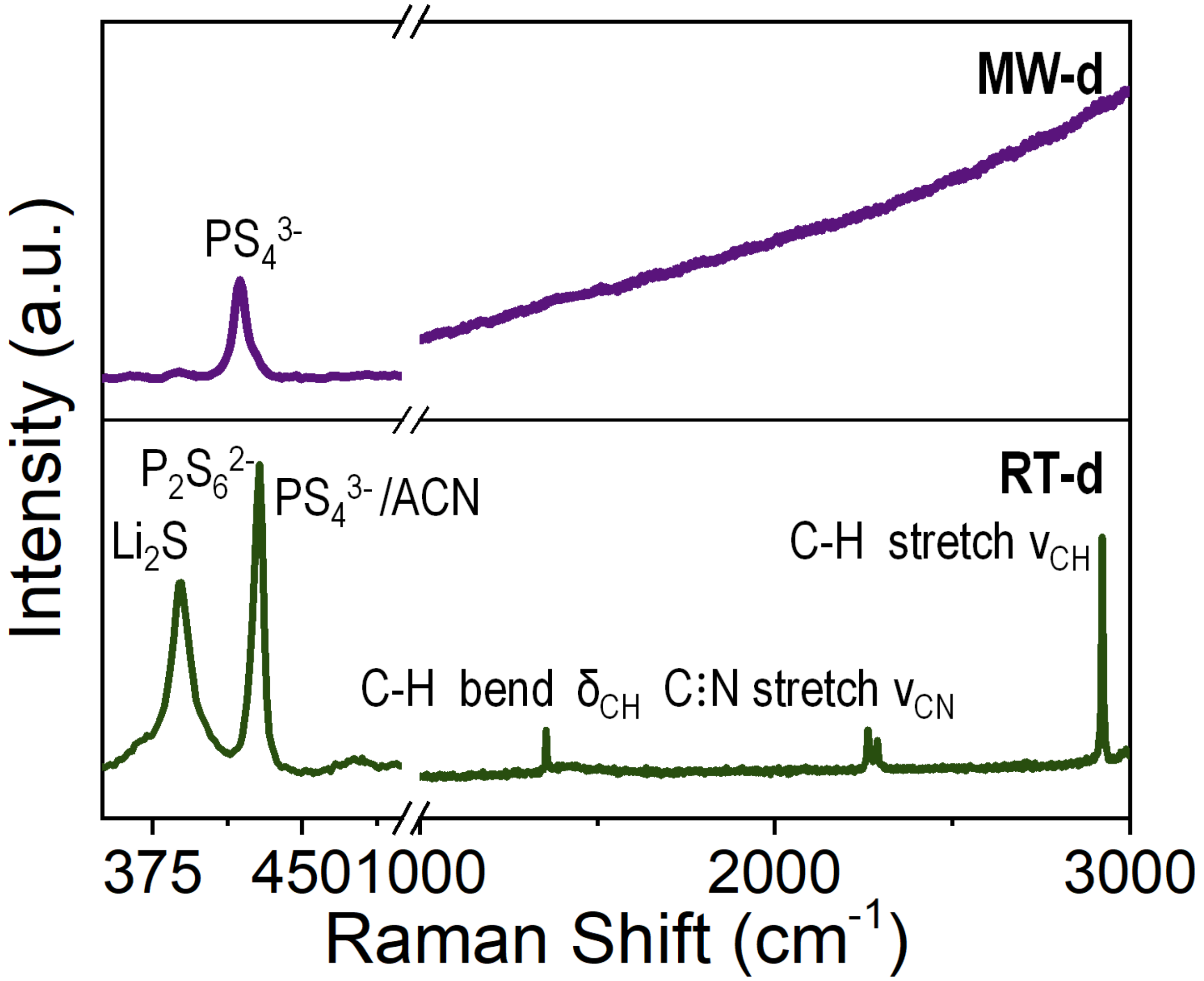

Figure S3. Raman spectra of dried LPSC precursor, which was synthesized through the MW-process (MW-d, top) and RT-process (RT-d, bottom)

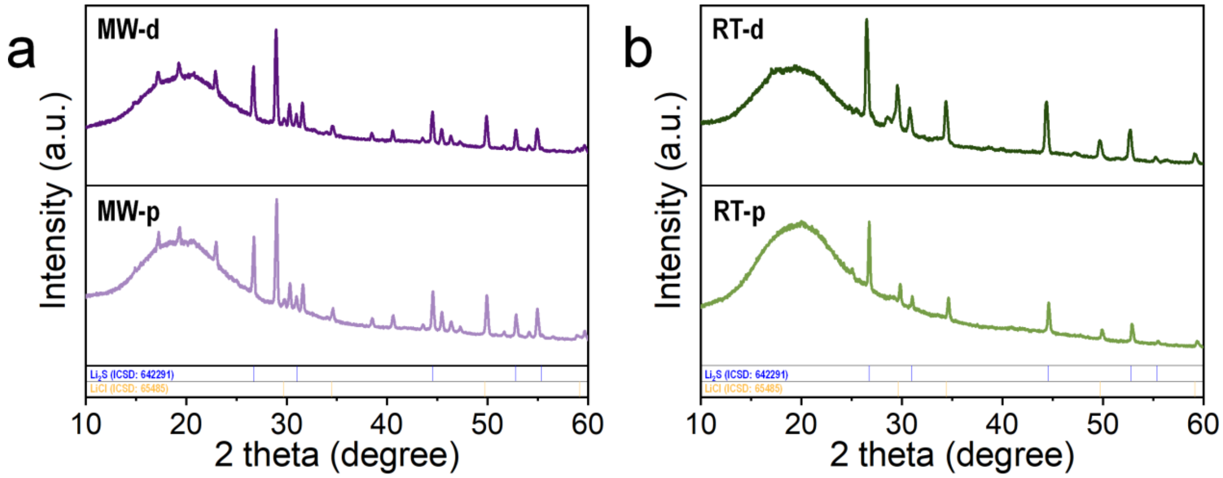

Figure S4. Crystallographic variation during the synthetic process. (a) XRD patterns of MW-d (top) and as-synthesized LPSC precursor that was prepared through the MW-process (MW-p) (bottom). (b) XRD patterns of RT-d (top) and as-synthesized LPSC precursor that was prepared through the RT-process (RT-p) (bottom).

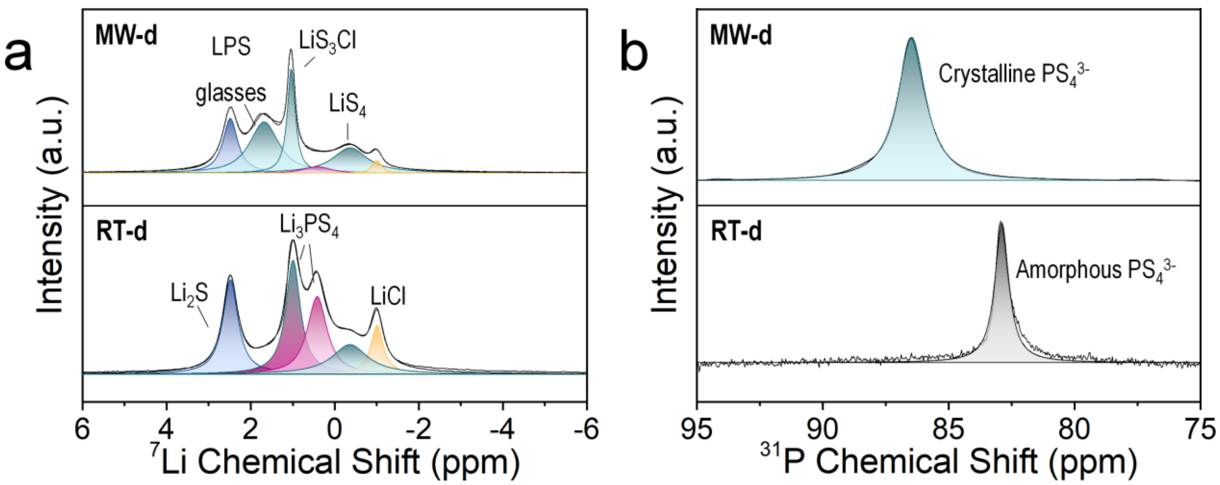

Figure S5. Solid-state MAS NMR results after drying process. (a) ^{7}Li MAS NMR spectra of MW-d (top) and RT-d (bottom). (b) ^{31}P MAS NMR spectra of MW-d (top) and RT-d (bottom).

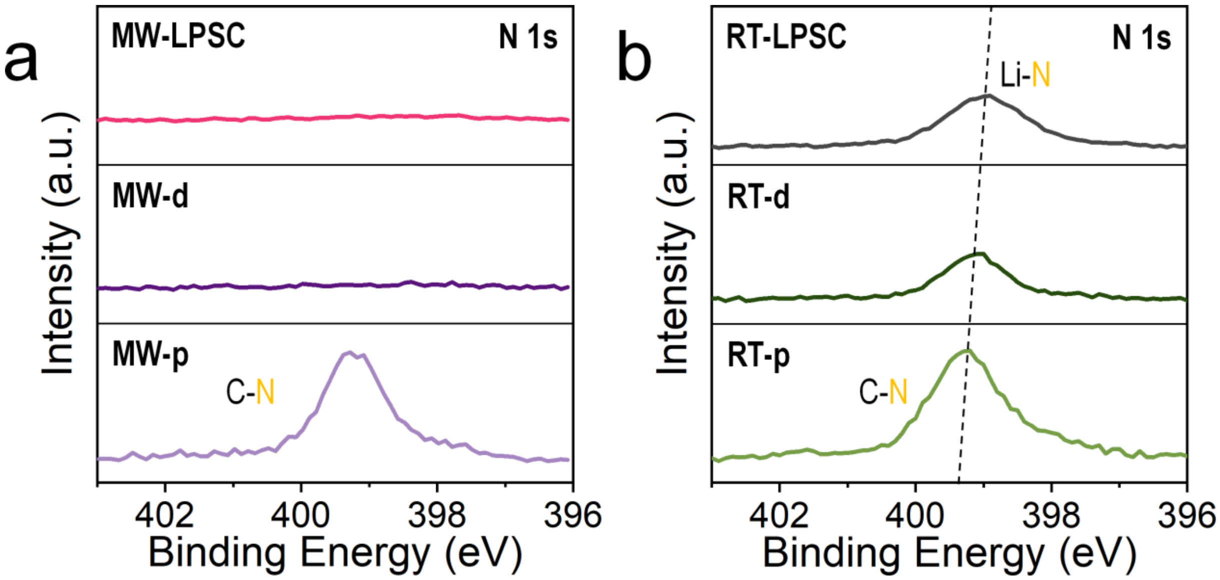

Figure S6. Effect of nucleophilic interaction of solvent in Li-argyrodite synthesis. (a) N 1s XPS spectra of MW-p (bottom), MW-d (middle), and Li-argyrodite LPSC that was synthesized through the MW-process (MW-LPSC) (top). (b) N 1s XPS spectra of RT-p (bottom), RT-d (middle), and Li-argyrodite LPSC that was synthesized through the RT-process (RT-LPSC) (top).

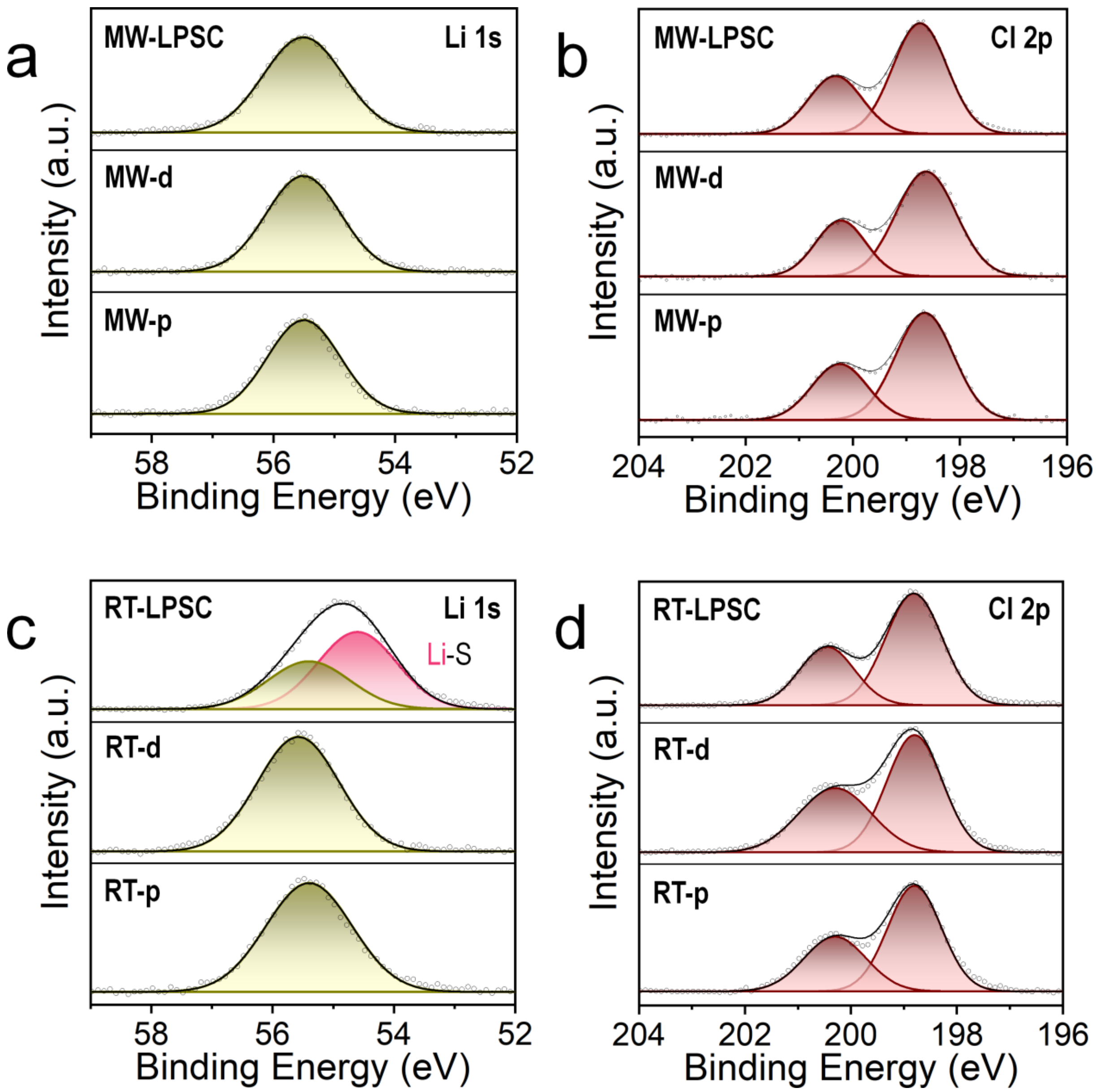

Figure S7. XPS spectra during synthesis. (a) Li 1s and (b) Cl 2p spectra of MW-p (bottom), MW-d (middle), and MW-LPSC (top). (c) Li 1s and (d) Cl 2p spectra of RT-p (bottom), RT-d (middle), and RT-LPSC (top).

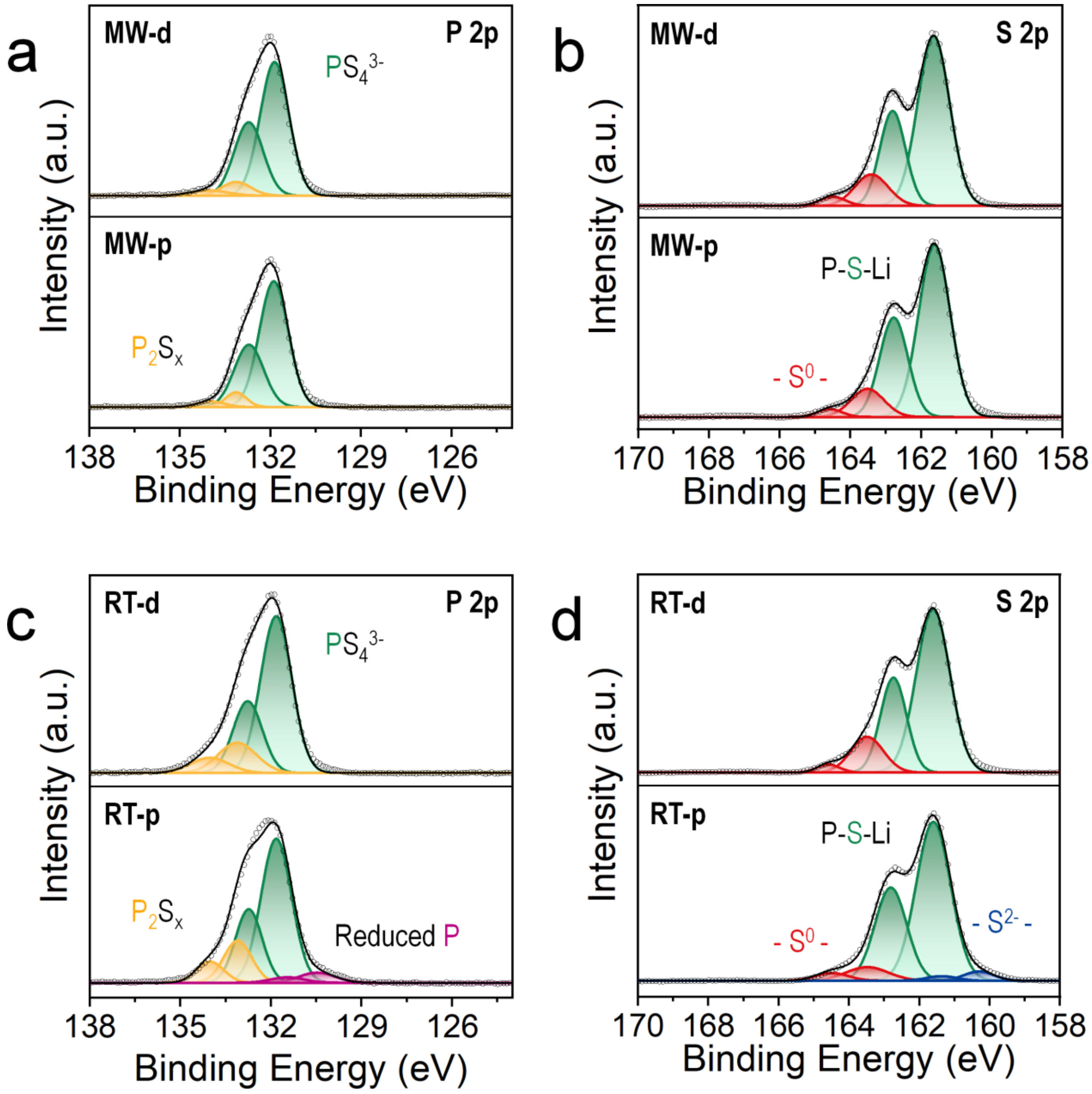

Figure S8. XPS characterization during the synthesis process. (a) P 2p and (b) S 2p spectra of MW-d (top) and MW-p (bottom). (c) P 2p and (d) S 2p spectra of RT-d (top) and RT-p (bottom).

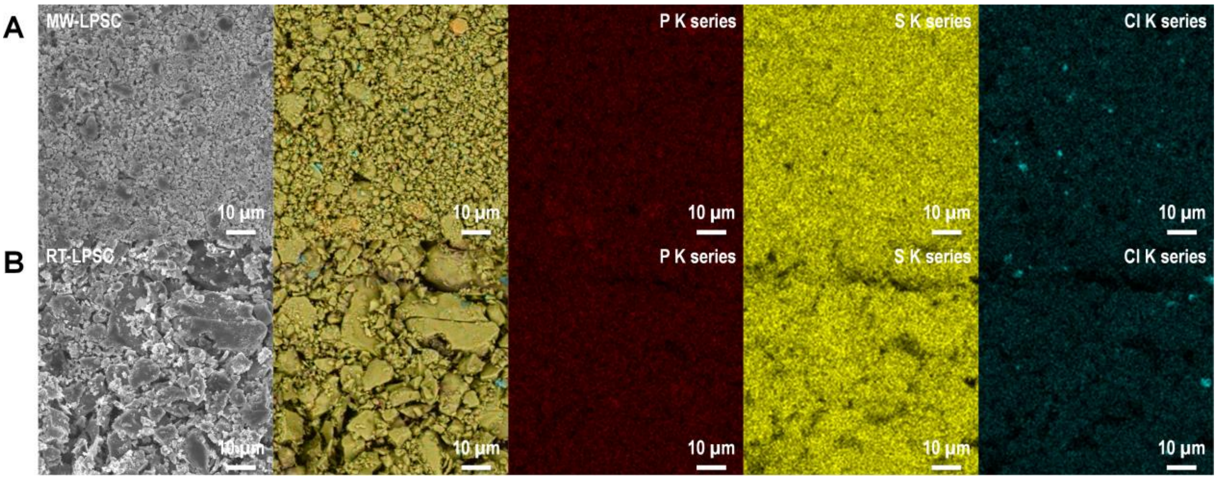

Figure S9. Morphological and elemental characterization of LPSC. (a and b) FESEM, backscattered electron mode image in FESEM along with layered EDS map, and EDS element mapping images of (a) MW-LPSC and (b) RT-LPSC.

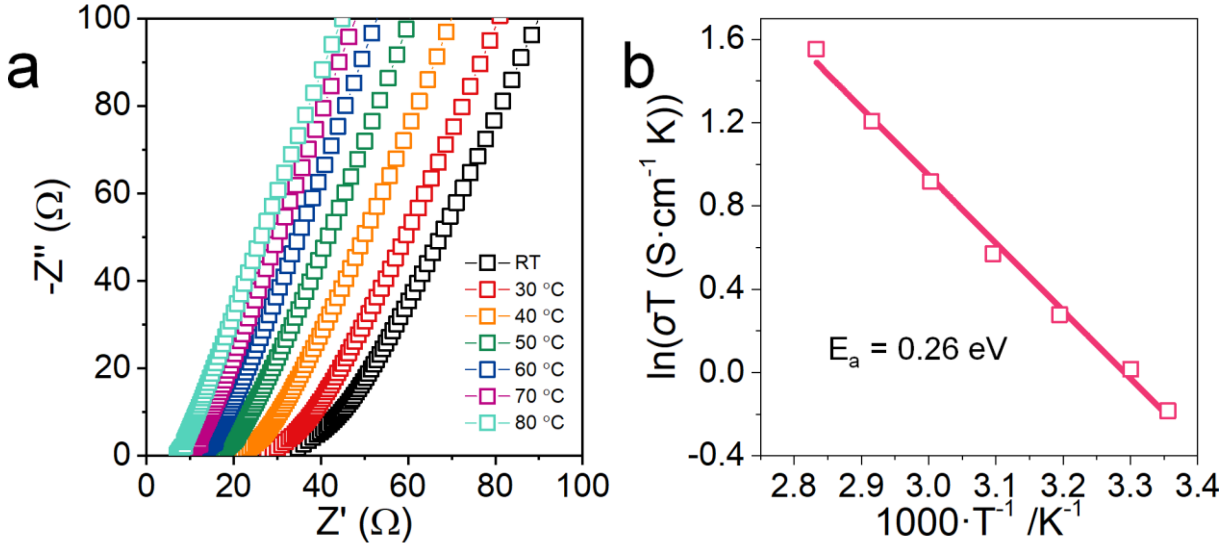

Figure S10. Temperature-dependent measurement results for evaluating the activation energy of MW-LPSC SEs. (a) Nyquist plot and (b) Arrhenius plot of MW-LPSC SEs in frequency range from 3 MHz to 10 mHz at temperature range from 25°C to 80°C.

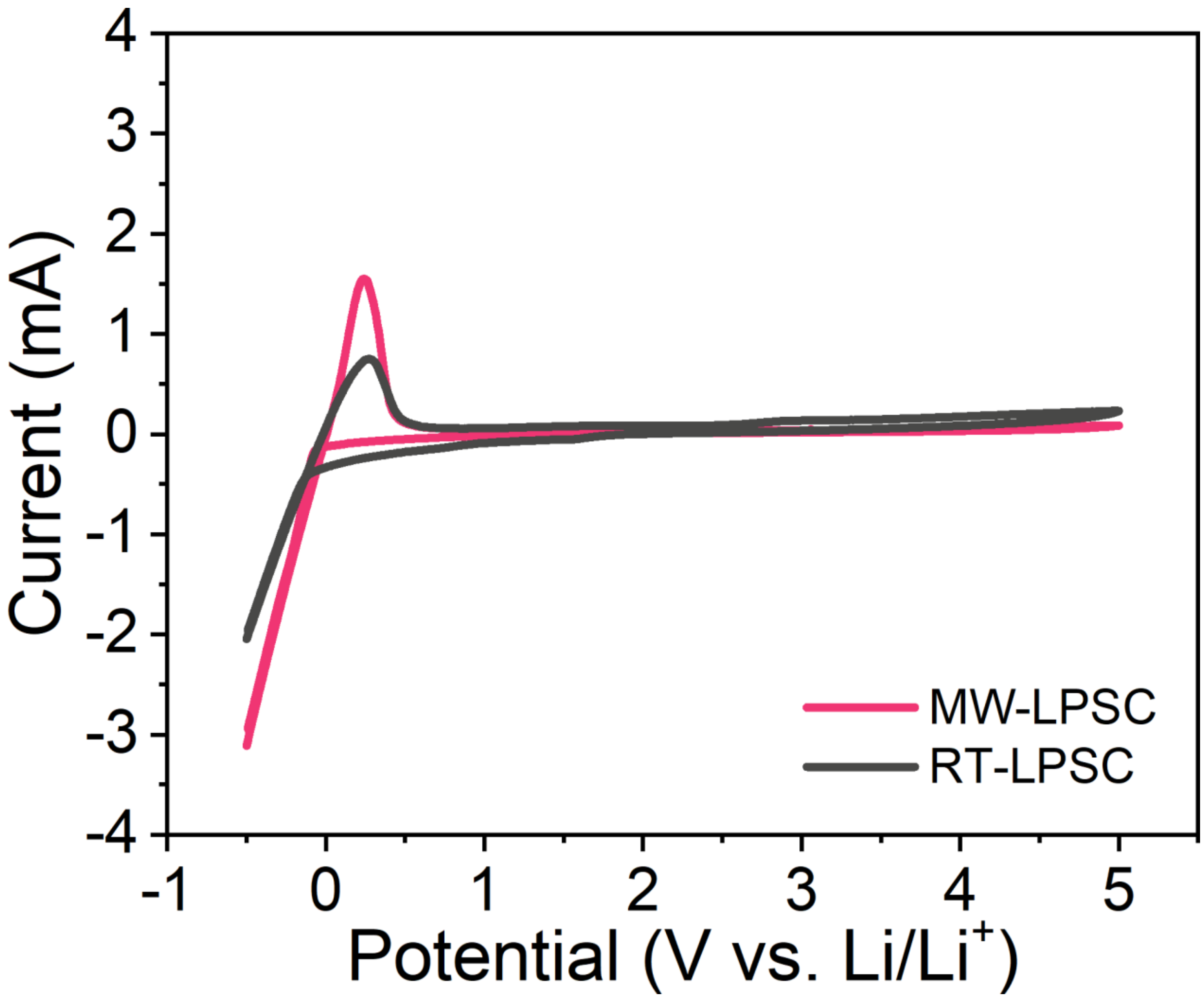

Figure S11. CV profiles of the MW-LPSC (red) and RT-LPSC (grey) in asymmetric cell with voltage range from -0.5 and 5 V at a scan rate of 5 mV s^{-1}.

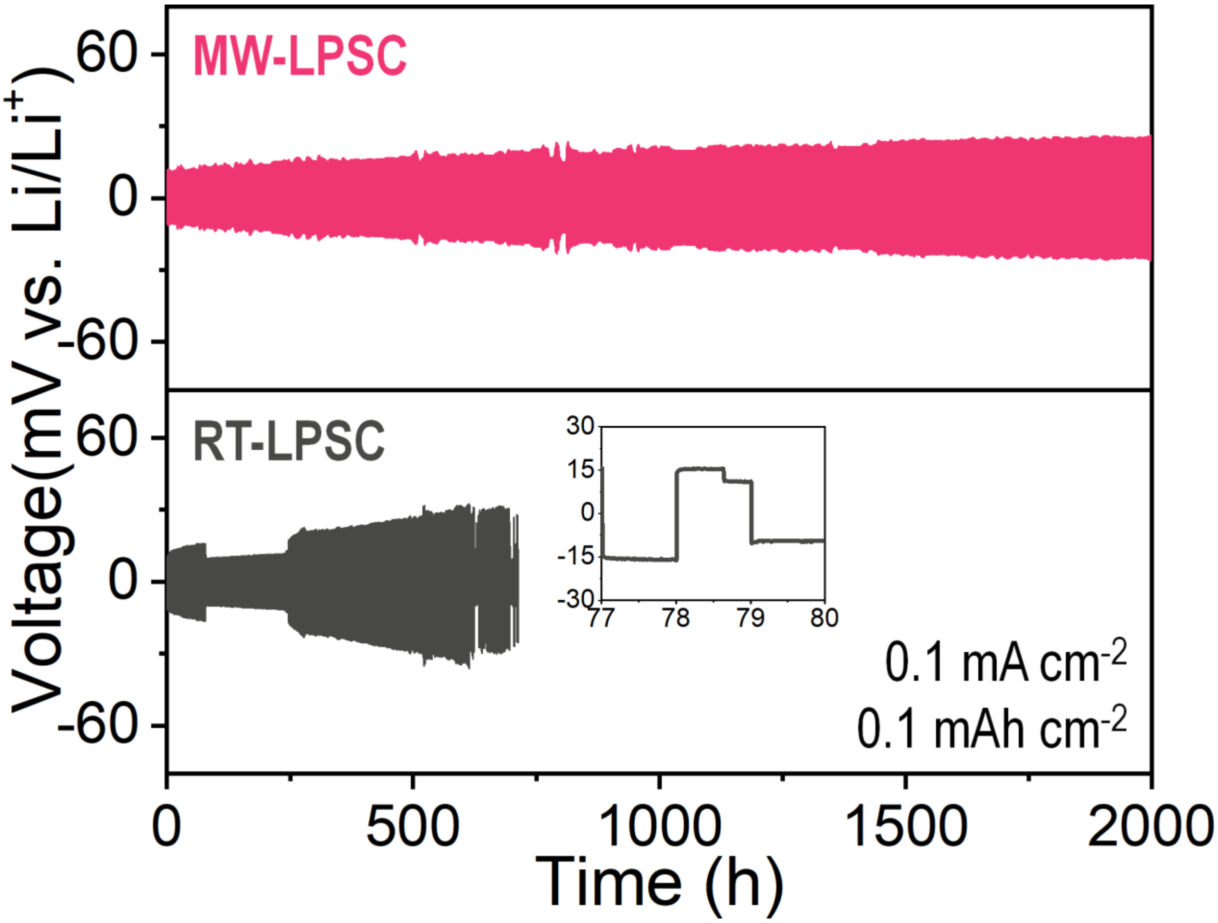

Figure S12. Voltage profile of galvanostatic charge/discharge Li|SEs|Li symmetric cell with various SEs: MW-LPSC (pink) and RT-LPSC (gray), cycled at current of 0.1 mA cm^{-1} and capacity of 0.1 mAh cm^{-2}.

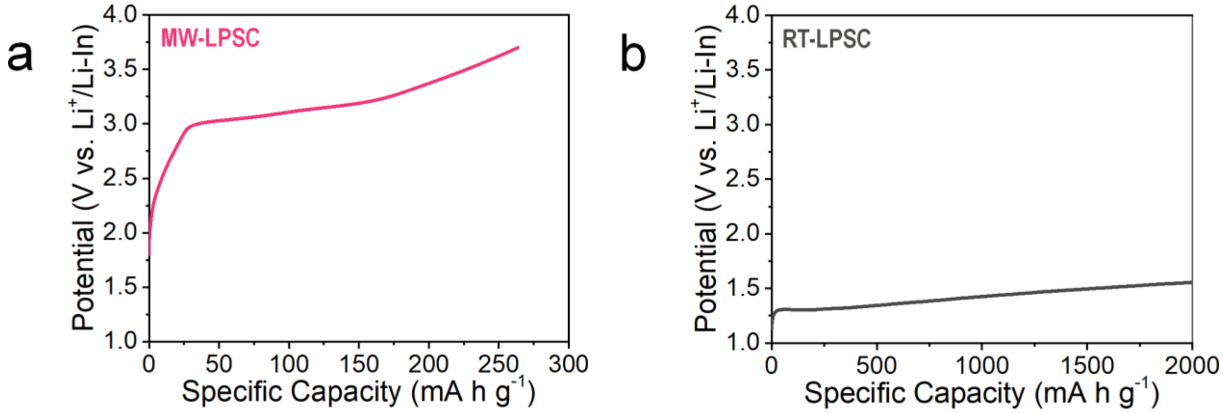

Figure S13. Load curves of the first charge profiles of the ASSBs with various SEs. (A) MW-LPSC and (B) RT-LPSC at 0.1 C.

Supplemental tables

Table S1. Refined parameters for MW-LPSC.

**Space group = F-43m, a = 9.84816 (18), R_{bragg} = 2.619**						
Atom	Wyckoff Site	x	y	z	Occ.	B_{eq}
Li1	48h	0.1737(9)	0.1737(9)	0.0065(12)	0.675(14)	5.259
P1	4b	0.5	0.5	0.5	1	1.429
S1	16e	0.62060(19)	0.62060(19)	0.62060(19)	1	2.448
Cl1	4d	0.25	0.25	0.25	0.37(9)	3.016
S2	4d	0.25	0.25	0.25	0.63(9)	3.016
Cl2	4a	0	0	0	0.63(9)	3.024

Table S2. Refined parameters for RT-LPSC.

Space group = F-43m, a = 9.85501 (5), R_{bragg} = 3.094						
Atom	Wyckoff Site	x	y	z	Occ.	B_{eq}
Li1	48h	0.1718(9)	0.1718(9)	0.0087(11)	0.604(10)	5.259
P1	4b	0.5	0.5	0.5	1	1.429
S1	16e	0.62052(14)	0.62052(14)	0.62052(14)	1	2.448
Cl1	4d	0.25	0.25	0.25	0.45(7)	3.016
S2	4d	0.25	0.25	0.25	0.55(7)	3.016
Cl2	4a	0	0	0	0.55(7)	3.024

Table S3. Resistances obtained from impedance fitting for RT-LPSC SEs.

**RT-LPSC**	**Resistance (ohm)**	**Specific Conductivity (mS cm^{-1})**
R_i	57	1.6
R_{gb}	4916	1.87 x 10^{-2}
R_e	38603	2.38 x 10^{-3}

Table S4. Comparison of electrochemical performances of ASSBs with Li-argyrodite SEs via wet-chemical synthesis using layered-structured cathodes.

Composition	Solvent	Ionic Conductivity (mS cm^{-1}) at 25 °C	Capacity (mAh g^{-1} @ 0.1 C) at 25 °C	Active Materials	Ref.
Li_6PS_5Cl	Ethyl acetate	1.1	145.8	$LiNi_{0.6}Co_{0.2}Mn_{0.2}O_2$	[5]
Li_6PS_5Cl	THF	2.03	156	$LiNi_{0.6}Co_{0.2}Mn_{0.2}O_2$	[6]
Li_6PS_5Cl	Ethanol	0.21	62 @ 0.064 mA cm^{-2}	$LiCoO_2$	[7]
Li_6PS_5Cl	THF	1.8	185.6 at 55 °C	$LiNi_{0.6}Co_{0.2}Mn_{0.2}O_2$	[8]
$Li_{5.8}PS_{4.8}Cl_{1.2}$	Ethylenediamine	2.87	175.7	$LiNi_{0.9}Co_{0.1}Mn_{0.1}O_2$	[9]
Li_6PS_5Br	THF /Ethanol	1.4	150 @ 0.13 mA cm^{-2}	$LiNi_{0.33}Co_{0.33}Mn_{0.33}O_2$	[10]
Li_6PS_5Br	Ethyl propionate /Ethanol	3.4 x 10^{-2}	109 @ 0.13 mA cm^{-2}	$LiNi_{0.33}Co_{0.33}Mn_{0.33}O_2$	[11]

References

[1] a)T. K. Zakharchenko, A. I. Belova, A. S. Frolov, O. O. Kapitanova, J. J. Velasco-Velez, A. Knop-Gericke, D. Vyalikh, D. M. Itkis, L. V. Yashina, *Top. Catal.* **2018**, 61, 2114; b)B. Fleutot, B. Pecquenard, H. Martinez, M. Letellier, A. Levasseur, *Solid State Ionics* **2011**, 186, 29.

[2] M. Ghidiu, R. Schlem, W. G. Zeier, *Batter. Supercaps* **2021**, 4, 607.

[3] K. Suto, P. Bonnick, E. Nagai, K. Niitani, T. S. Arthur, J. Muldoon, *J. Mater. Chem. A* **2018**, 6, 21261.

[4] J. C. Deàk, L. K. Iwaki, D. D. Dlott, *J. Phys. Chem. A* **1998**, 102, 8193.

[5] S. Choi, J. Ann, J. Do, S. Lim, C. Park, D. Shin, *J. Electrochem. Soc*. **2018**, 166, A5193.

[6] Y. J. Heo, S. D. Seo, S. H. Hwang, S. H. Choi, D. W. Kim, *Int. J. Energy Res*. **2022**, 46, 17644.

[7] Z. X. Zhang, L. Zhang, Y. Y. Liu, X. L. Yan, B. Xu, L. M. Wang, *J. Alloy Compd.* **2020**, 812, 152103.

[8] M. J. Kim, I. H. Choi, S. C. Jo, B. G. Kim, Y. C. Ha, S. M. Lee, S. Kang, K. J. Baeg, J. W. Park, *Small Methods* **2021**, 5, 2100793.

[9] Y. Subramanian, R. Rajagopal, K. S. Ryu, *Scr. Mater.* **2021**, 204, 114129.

[10] S. Yubuchi, M. Uematsu, C. Hotehama, A. Sakuda, A. Hayashi, M. Tatsumisago, *J. Mater. Chem. A* **2019**, 7, 558.

[11] S. Chida, A. Miura, N. C. Rosero-Navarro, M. Higuchi, N. H. H. Phuc, H. Muto, A. Matsuda, K. Tadanaga, *Ceram. Int.* **2018**, 44, 742.
